# Refractory necrotizing scleritis successfully treated with adalimumab

**DOI:** 10.1186/s12348-016-0107-y

**Published:** 2016-10-12

**Authors:** Lola E. Lawuyi, Avinash Gurbaxani

**Affiliations:** 1 Gate 17, Villa 970 Al Raha Gardens, Abu Dhabi, United Arab Emirates; 2 Moorfields Eye Hospital Dubai, Medical Retinal and Cataract, Dubai, United Arab Emirates

**Keywords:** Necrotizing scleritis, TNF inhibitors, Adalimumab

## Abstract

Necrotizing scleritis is the most severe and destructive form of scleritis with vision-threatening sequelae. It is divided into with inflammation and without inflammation (scleromalacia perforans). Adalimumab is a tumour necrosis factor (TNF)-inhibiting anti-inflammatory medication licensed for the treatment of rheumatoid and psoriatic arthritis, ankylosing spondylitis and inflammatory bowel disease (in the USA). We report two cases of necrotizing scleritis successfully treated with adalimumab.

## Case report

### Case 1

A 40-year-old lady presented with a history of scleritis for which she was on oral prednisolone for over 1 year with doses ranging from 10 to 80 mg per day. She had pain and discomfort. Her visual acuity was 6/6 and posterior segment was normal. Examination revealed an area of scleral necrosis.

As her scleritis was active, she was commenced on prednisolone 80 mg and mycophenolate mofetil (MMF) (Cellcept) 500 mg BD, increasing to 750 mg BD. However, she had severe gastric discomfort and nausea and was unable to continue with MMF. She was subsequently put on tacrolimus (Prograf) 7.5 mg but developed nausea and lethargy on this. She was unable to reduce oral steroids whilst oral immunosuppression was tried. She was then started on adalimumab (Humira) subcutaneous 40 mg fortnightly and methotrexate 12.5 mg OD and achieved resolution and control of scleral necrosis after 4 weeks. During the course of treatment, she developed one episode of gastroenteritis during which she missed her Humira. At 9-month follow-up, the disease has remained quiet on two weekly subcutaneous adalimumab treatments.

### Case 2

**Fig. 1:**
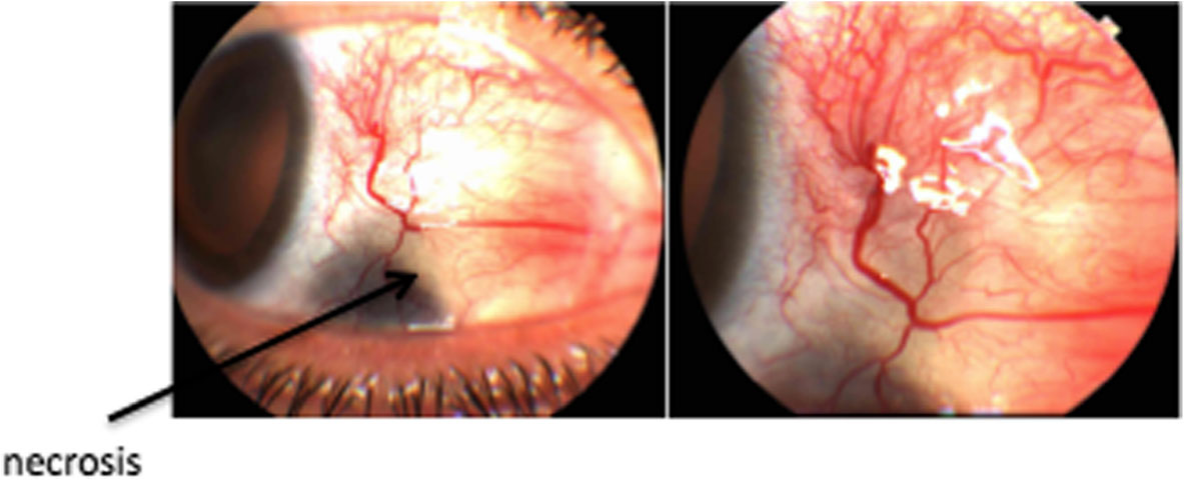
Scleral necrosis RE

**Fig. 2:**
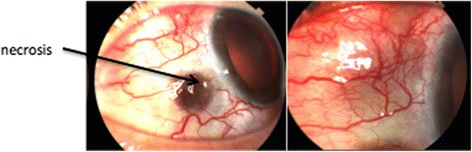
Scleral necrosis LE

A 52-year-old lady with known rheumatoid arthritis and refractory necrotizing scleritis poorly controlled with oral prednisolone 10–80 mg and oral methotrexate 15 mg once a week was referred for treatment. Her visual acuity was 6/9 in both eyes. Slit lamp examination revealed acute scleral necrosis and impending perforation as shown in Figs. 1 and 2.

**Fig. 3:**
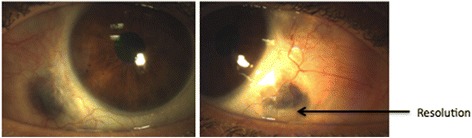
Resolution/control on adalimumab at 6-month follow-up

Her past medical history included one episode of scleral perforation to the left eye treated with amniotic patch graft. The patient was started on two weekly subcutaneous adalimumab 40 mg and achieved resolution and control of scleral necrosis after 8 weeks. At 6-month follow-up, the disease has remained quiet on two weekly subcutaneous adalimumab treatments (Fig. 3).

Side effects during treatment included one episode of shingles.

## Discussion

Necrotizing scleritis represents 10–15 % of cases of anterior scleritis and is the most severe [1]. Left untreated, necrotizing scleritis has a rapid and destructive course that can be sight threatening.

Conventional treatment for recurrent or severe cases of necrotizing scleritis involves immunosuppression therapy and high-dose oral steroids [2]. Side effects associated with these treatments and a poor response may require patients to terminate treatment (as did our two patients), making this condition very difficult to manage.

The predominant cytokine TNF-alpha induces the matrix metalloproteinases (MMPs) enzymes, MMP-3 and MMP-9 [3]. These are predominantly responsible for the destruction of the scleral wall and adjacent cornea in severe scleritis, particularly associated with necrosis [4].

Infliximab (Remicade Centocor, Johnson & Johnson, USA) and adalimumab (Humira, Abbott Laboratories, Chicago, IL) are monoclonal antibodies that recognise, bind to and inhibit TNF, thereby decreasing inflammation.

Infliximab has shown long-term efficacy in the treatment of refractory posterior uveitis [5]. Published literature has reported improvement in patients with either idiopathic scleritis or scleritis associated with systemic disease treated with infliximab [6].

Ragam et al. evaluated the use of TNF inhibitors infliximab and adalimumab in 17 patients with non-infectious and non-necrotizing scleritis and achieved control of active inflammation for at least 2 months in 15 (88 %) of 17 patients [7].

Adalimumab has been reported as successful in the treatment of bilateral idiopathic nodular scleritis (with no side effects reported) [8] and nodular scleritis associated with RA [9].

Morarji et al. reported the successful treatment of necrotizing scleritis (secondary to granulomatosis with polyangiitis) with infliximab whilst waiting for rituximab to induce disease remission [10]. Two intravenous 1-g doses of rituximab (MabThera, Hoffmann-La Roche, Ltd.) (a chimeric murine and human monoclonal antibody directed against the CD20 antigen [11]) were used, given 2 weeks apart. However after 3 weeks, the necrotizing scleritis was still active. Therefore, infusions of infliximab (Remicade, Merck & Co.) 5 mg/kg doses at baseline, 2, 6 and 12 weeks were given, controlling the necrotizing scleritis by week 6. This enabled cyclophosphamide to be avoided in the acute phase (as rituximab can take up to 6 months to work).

In this case series, we report two cases of refractory necrotizing scleritis that were successfully treated with adalimumab. During treatment, one patient developed gastroenteritis and the other developed shingles.

Known potential side effects have been documented in clinical trials and postmarket surveillance with TNF inhibitor use. The development of injection site reactions [12], infusion reactions [12], and the reactivation of latent tuberculosis [13] is increased with TNF inhibitor use. Therefore, proper screening of patients with the tuberculin skin test and chest x-ray and/or interferon gamma assay should be performed prior to initiating these therapies. Other side effects include the development of demyelinating disease [14] and heart failure [15].

In conclusion, despite the risks involved with TNF inhibitor use, with adequate screening of patients, the present case series highlights that adalimumab offers a new tool for the treatment of refractory necrotizing scleritis when conventional treatments are contraindicated or have failed.
